# The role of intermittent continuous glucose monitoring in a successful outpatient transition from insulin to glibenclamide in a patient with transient neonatal diabetes

**DOI:** 10.20945/2359-3997000000484

**Published:** 2022-05-25

**Authors:** Arthur Lyra, Fernanda Rodrigues Ferreira, Regina Célia Santiago Moisés, Renata Maria de Noronha, Luis Eduardo Calliari

**Affiliations:** 1 Unidade de Endocrinologia Pediátrica Departamento de Pediatria Faculdade de Ciências Médicas da Santa Casa de São Paulo São Paulo SP Brasil Ambulatório de Diabetes, Unidade de Endocrinologia Pediátrica, Departamento de Pediatria, Faculdade de Ciências Médicas da Santa Casa de São Paulo, São Paulo, SP, Brasil; 2 Universidade Federal de São Paulo Escola Paulista de Medicina Divisão de Endocrinologia SP Brasil Divisão de Endocrinologia, Escola Paulista de Medicina, Universidade Federal de São Paulo, SP, Brasil

## Abstract

Neonatal diabetes mellitus (NDM) is a monogenic form of diabetes occurring mainly in the first 6 months of life. Approximately 30% of transient NDM (TNDM) cases will have an activating mutation in the K_ATP_ channel genes *ABCC8* and *KCNJ11*. The majority of the patients with *KCNJ11* mutations who are receiving insulin treatment can be transferred to treatment with sulfonylurea (SU), with an improvement in metabolic control and quality of life. Intermittent continuous glucose monitoring (iCGM) is used to assess the current and retrospective interstitial glucose, providing information such as hypo/hyperglycemia tendency and time on target. This case report describes the use of iCGM in the transition from insulin treatment to glibenclamide in a patient with TNDM caused by a pathogenic variant of *KCNJ11*. This is the first report of a successful outpatient transition from insulin to glibenclamide, in a Brazilian child with TNDM using iCGM (FreeStyle Libre@). The remote monitoring and online management allowed the patient to safely stay at home during the transition from insulin to SU, especially important in the context of the COVID-19 pandemic. We conclude that iCGM is a helpful tool in cases of NDM and should be used to increase safety and speed up dose adjustments in outpatient transition from insulin to glibenclamide.

## INTRODUCTION

Neonatal diabetes mellitus (NDM), a rare condition with an estimated incidence of 1:90,000-210,000 live births (1), is a monogenic disorder with onset mainly in the first six months of life (1). It can be permanent (PNDM), where lifelong treatment is required, or transient (TNDM), where remission occurs within months with recurrence in approximately half of the patients later in life (1-3).

Approximately 30% of TNDM cases will have an activating mutation in the K_ATP_ channel genes *ABCC8* and *KCNJ11*, which code, respectively, for the SUR1 and Kir6.2 subunits of the channel (4). Pathogenic gain-of-function variants in the *KCNJ11* gene cause the permanent opening of the K_ATP_ channel, which maintains the resting state of the cell membrane of the beta-cell. Thus, there is no depolarization of the cell membrane, preventing insulin release (5)

The literature shows that most patients with *KCNJ11* pathogenic variants being treated with insulin could be transferred to treatment with sulfonylurea (SU). The transfer to SU is usually done in the hospital to guarantee the patient’s safety, and the recommendation is to measure glucose before and two hours after each meal, at bedtime, and 2AM (6,7). To perform this transition at home, families require training regarding monitoring and management of hypoglycemia.

Continuous glucose monitoring (CGM) systems, either real-time or intermittent (iCGM), have recently become more widely available. The iCGM assesses current and retrospective interstitial glucose and comprises: 1. A glucose sensor attached to the patient’s arm; 2. A reader, which receives data and evaluates time in target range, as well as hypo/hyperglycemia tendencies. These devices could potentially offer support in the transition from insulin to sulfonylurea therapy in NDM (8).

Here, we report the case of a patient with TDNM due to a mutation in the *KCNJ11* gene, where the use of iCGM was found to be an important tool during the transition from insulin to SU in outpatient care, during the context of the COVID-19 pandemic.

## CASE REPORT

We report a 6.8 year-old Brazilian boy, who is the second child of a non-consanguineous Caucasian couple, with no family history of diabetes mellitus. He was born at 38 weeks gestation, birth weight of 2,185 g, and birth length of 44cm (both below -2SDS).

At five months of age, he presented with respiratory symptoms (dyspnea) and was reviewed in the emergency room. He had a blood glucose of 600 mg/dL, acidosis, and ketonuria and insulin therapy was initiated. After four days, he was discharged using insulin glargine 2 IU/day and referred to our Pediatric Endocrine Unit.

Further investigation revealed glycated hemoglobin (HbA1c) of 8.4%, undetectable C-peptide, negative islet-cell antibody, glutamic acid decarboxylase antibody and insulin auto-antibody. The diagnosis of NDM was suspected and molecular genetic evaluation was performed. A missense heterozygous mutation p.Glu227Lys (c.679G>A) was found in the KCNJ11 gene, on chromosome 11p15.1. The E227K mutation was previously reported as a pathogenic variant (6). The parents are asymptomatic and were not tested for this mutation.

During follow-up, his insulin requirements gradually decreased, and insulin therapy ceased at the age of 1.2 years. In the following five years after insulin discontinuation, the patient remained asymptomatic, his HbA1c levels ranged from 6.1-6.5% and C-peptide was 0.8 ng/mL.

At 6.5-years-old, polydipsia and polyuria were noticed by his parents. He was brought in for an evaluation, his weight was 25.6 kg (+1.2 SDS), height 112.5 cm (-1.2 SDS), and BMI 20.2 kg/m^2^ (+2.5 SDS). Laboratory tests showed HbA1c: 8.5%, fasting blood glucose: 177 mg/dL and C-peptide: 1.1 ng/mL, indicating a relapse of diabetes.

He was therefore re-started on insulin, on November 28^th^ 2020. The initial dose was degludec 1 IU/day and fast-acting insulin lispro 0.5IU before meals if hyperglycemic.

**Figure 1:**
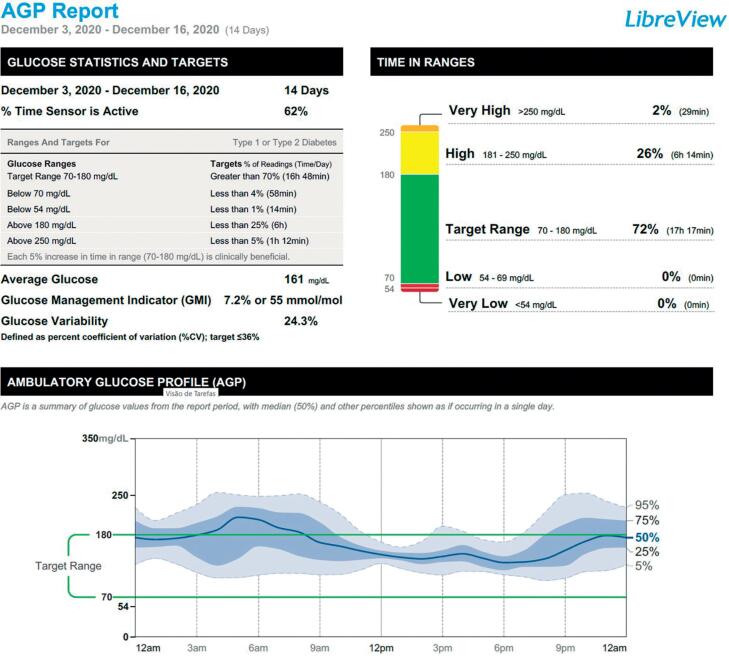
Two-week iCGM report using insulin.

After insulin was initiated, the use of iCGM (FreeStyle Libre@) was proposed and accepted by the family. This system is approved by the National Health Surveillance Agency for children aged 4 years and over. During insulin therapy, the device’s 14-day analysis revealed a glucose management index (GMI) of 7.2%, 72% of the time in range (70-180 mg/dL), 28% above target, and 0% hypoglycemia (Figure 1).

After glucose stabilization it was decided to switch therapy from insulin to a SU (glibenclamide). This switch is done in the hospital in the majority of patients (7) to ensure a safe transition, but given the restrictions due to the COVID-19 pandemic, it was decided that the patient should undertake the transition as an outpatient. During transition data from iCGM was uploaded using an online platform (LibreView platform@). Access to this website allowed the medical team to perform remote daily checks on glucose levels variations and suggest treatment changes.

**Figure 2:**
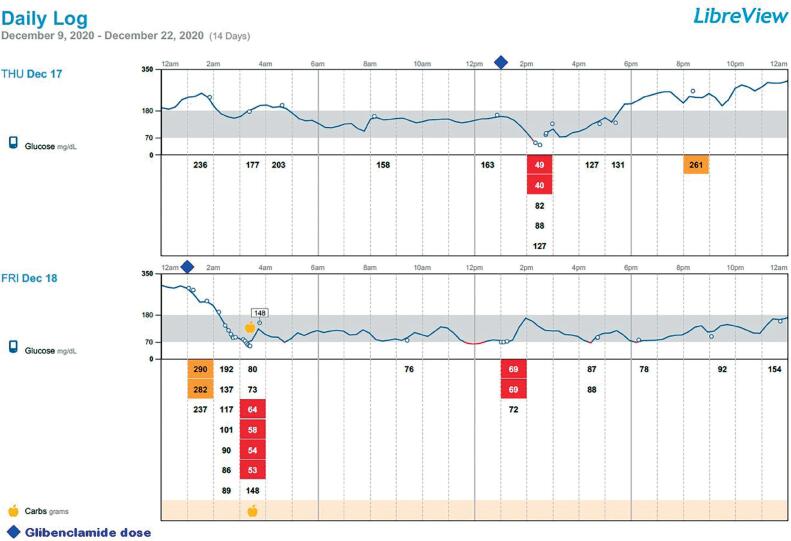
First two days of therapy with glibenclamide (SU) at 1 pm and 1 am.

Oral glibenclamide was started at a dose of 2.5 mg every 12 hours (0.2 mg/kg/day) on December 17^th^ 2020. The patient’s glucose profile improved rapidly, and insulin therapy was discontinued on the second day (Figure 2).

**Figure 3:**
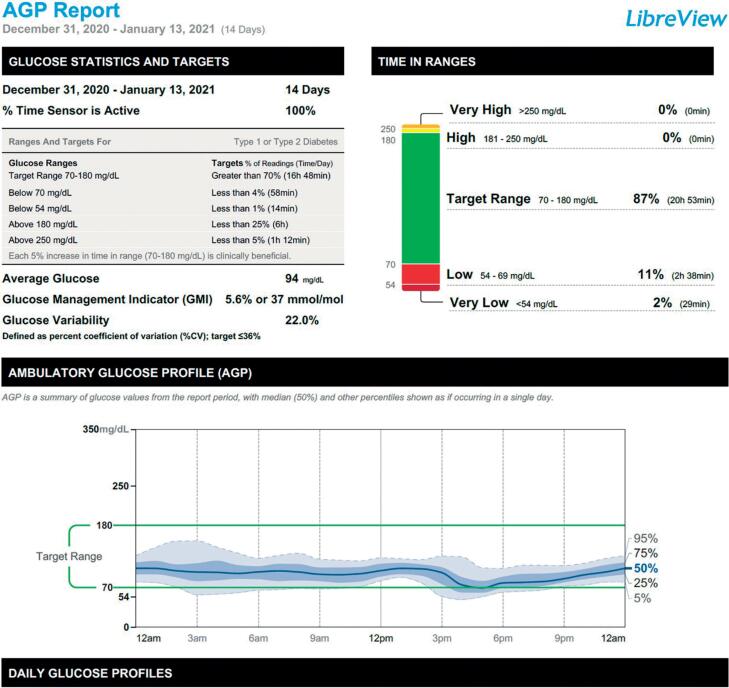
Ambulatory glucose profile (AGP) report during the first days of starting treatment with SU.

Sensor data guided changes in glibenclamide dose, which was gradually reduced to a final dose of 1.25 mg once daily (0.05 mg/kg/day). After 44 days of SU treatment, sensor data showed a time in range of 87%, GMI of 5.6% (Figure 3) and a decrease of HbA1c to 7.1%.

**Figure 4:**
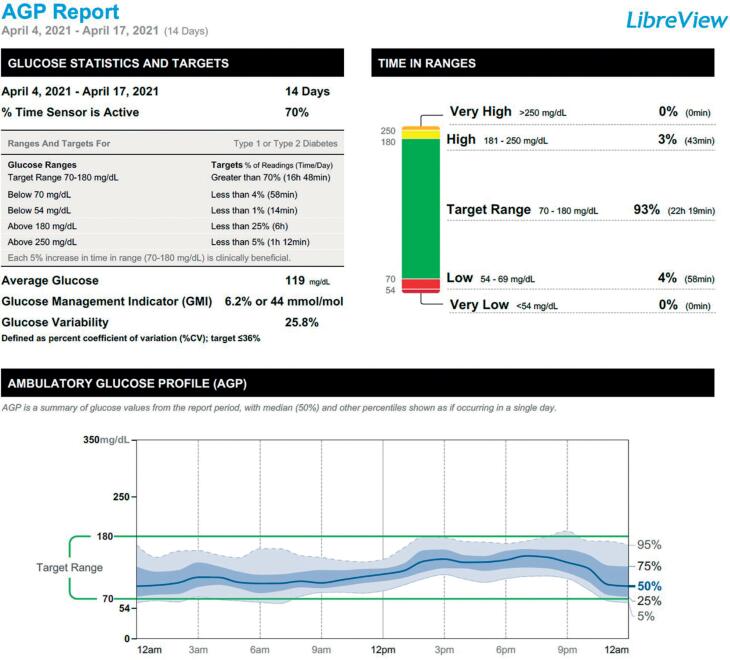
iCGM reports after 4 months of SU treatment.

After four months of SU treatment, HbA1C level was 6,1% and iCGM showed that the patient had low glycemic variability, GMI of 6.2%, 93% of the time in the target range, 3% of the time in hyperglycemia, 4% hypoglycemia (54-69 mg/dL) (Figure 4).

## DISCUSSION

We present a case where the use of precision medicine and technology support allowed a successful transition from insulin to glibenclamide without hospitalization in a challenging period during COVID-19 pandemic. Our patient had a diagnosis of NDM and absence of autoantibodies (2). He was born small for gestational age, which is found in 62% of cases of NDM (2). Genetic testing is strongly recommended for neonates with persistent hyperglycemia (9). The pathogenic variant found in the *KCNJ11* gene, E227K is a gain-of-function mutation that decreases sensitivity to ATP in the K_ATP_ channel, causing reduction in insulin secretion that evolves with TNDM (9). Our patient had a remission of diabetes at 30 weeks of age and a relapse around six years old. These periods are consistent with the published data on TNDM, where remission occurred with a mean age of 35 weeks (5-208) and relapse at 4.7 years (3-15) (10). A systematic review of 103 articles analyzed the transfer from insulin treatment to sulfonylurea in 502 cases of NDM, 82.3% as a result of mutations of the *KCNJ11* gene, and found a success rate of 91%. The average dose of SU required for the treatment of NDM was 0.48 mg/kg/day (0.017-2.8 mg/kg/day). The pathogenic variant E227K was described in 13 cases and was classified as being sensitive to treatment with SU since all cases responded to glibenclamide (6). In our patient, we reached a dose of 0.05 mg/kg/day, which is lower than the dose described in a meta-analysis with 81 patients with *KCNJ11* mutations (0.12-0.45 mg/kg/day). Bowman and cols. enrolled 81 NDM carriers of KCNJ11 gene mutations and reported 93% effectiveness of SU as a long-term monotherapy, with an average follow-up duration of 10.2 years. In addition, the safety of SU is highlighted in the study, with no reports of seizures or loss of consciousness caused by hypoglycemia (11). The 0.05 mg/kg/day dose is equivalent to ¼ of a tablet that the parents have to crush and dilute with water since there is no other option available in Brazil at present. The use of a SU suspension like Amglidia^®^, recently authorized in France, could, theoretically, enable the delivery of more accurate dosages (12). This medication is still not approved by the Brazilian Health Surveillance Agency.

To the best of our knowledge, this is the first report of a successful outpatient transition from insulin to glibenclamide with iCGM in a Brazilian child with TNDM. Two articles reported Brazilian patients with *KCNJ11* mutations where a transition to glibenclamide was attempted, the first in a child with PNDM without response (13), and the second in an adult patient with a PNDM who successfully transitioned (14).

The outpatient protocol published by Pearson and cols. (7) proposes that capillary blood glucose, performed using blood from lancet finger pricks, should be tested before all meals and at bedtime. As an alternative, we used iCGM for the measurement of interstitial glucose. With CGM, there was an increase in the number of glucose measurements to an average of 15 iCGM scans/day. This increase in measurements without invasive procedures helped to improve the distress caused by finger pricks and also gave more information regarding glucose variations during the transition phase.

The use of iCGM had another important role in this case. Due to the COVID-19 pandemic, hospitalizations were restricted to severe cases. The use of a system with an automatic real-time transmission of interstitial glucose values from the sensor to the LibreView platform@ allowed the medical team to monitor glucose every day during the transition period. This analysis enabled the identification of patterns of hyper/hypoglycemia daily, thus accelerating therapeutic decisions to optimize treatment and reduce risks for the patient. Dahl and Kumar also mention the importance of using CGM, especially due to the risk of hypoglycemia in children with NDM, whose signs can be difficult to recognize, as the infant can present nonspecific signs such as behavioral changes and irritability (9).

The advantages of using CGM associated with online data-sharing become more obvious when comparing this new method with the outpatient protocol proposition in which the physician should see the patient every week and be accessible by phone every day during the transfer (8).

Therefore, access to the iCGM information allowed the switch to SU to occur without hospitalization, minimizing the exposure of the family to in-hospital contamination, yet assuring safety by giving the medical team real-time results that were used to monitor and promptly adjust SU dosages (15). Rabbone and cols. describe the use of CGM (Medtronic Enlite/Dexcom G4) integrated with continuous subcutaneous insulin infusion pumps (CSII) therapy, which allows for more precise glycemic control. This article mainly focuses on how the use of CSII allows the gradual and safe substitution of insulin therapy for glibenclamide, reducing the risk of diabetic ketoacidosis (DKA) (16). The use of CSII may be considered the gold standard in infants with neonatal diabetes, but is still not widely available. The use of iCGM in patients on MDI could be both safe and affordable when CSII is not an option.

The use of the iCGM has its limitations – the device may suffer damage with use, fall from the patient’s arm or contain a calibration error. If any of these occurrences happen, the recommendation is to replace the sensor or start capillary blood glucose measurements. In addition, the family was instructed to confirm hypoglycemia identified by the iCGM through capillary blood glucose, as sensor displacement could generate low glucose values. In the case described none of these problems occurred.

The strengths of our report rely on the importance of the genetic diagnosis of TDM to define the best therapeutic option and the use of technological support (iCGM) to increase safety and convenience for the patient. The weakness is that this is just one case, not enough to generalize the indication of iCGM to help transition from insulin to SU to be done at home. Nonetheless, we believe that this experience can be used as an example and eventually replicated in other settings.

We conclude that neonatal diabetes is a model of a genetic disease that can benefit from precision medicine, where treatment is defined after molecular diagnosis, and that iCGM is a valuable tool that should be considered to monitor glucose during the process of SU introduction to these patients.
